# Fructose: a modulator of intestinal barrier function and hepatic health?

**DOI:** 10.1007/s00394-023-03232-7

**Published:** 2023-08-18

**Authors:** Raphaela Staltner, Katharina Burger, Anja Baumann, Ina Bergheim

**Affiliations:** Department of Nutritional Sciences, Molecular Nutritional Science, University of Vienna, Josef-Holaubek-Platz 2, A-1090 Vienna, Austria

**Keywords:** Sugar, Intestinal barrier, Tight junctions, Nitric oxide, Intestinal microbiota

## Abstract

**Purpose:**

Consumption of fructose has repeatedly been discussed to be a key factor in the development of health disturbances such as hypertension, diabetes type 2, and non-alcoholic fatty liver disease. Despite intense research efforts, the question if and how high dietary fructose intake interferes with human health has not yet been fully answered.

**Results:**

Studies suggest that besides its insulin-independent metabolism dietary fructose may also impact intestinal homeostasis and barrier function. Indeed, it has been suggested by the results of human and animal as well as *in vitro* studies that fructose enriched diets may alter intestinal microbiota composition. Furthermore, studies have also shown that both acute and chronic intake of fructose may lead to an increased formation of nitric oxide and a loss of tight junction proteins in small intestinal tissue. These alterations have been related to an increased translocation of pathogen-associated molecular patterns (PAMPs) like bacterial endotoxin and an induction of dependent signaling cascades in the liver but also other tissues.

**Conclusion:**

In the present narrative review, results of studies assessing the effects of fructose on intestinal barrier function and their impact on the development of health disturbances with a particular focus on the liver are summarized and discussed.

## Introduction

The idea that the intake of certain macronutrients like sugar may be linked to and even trigger the development of diseases is not a new concept. Already at the beginning of the last century, Emerson and Larimore reported a strong association of dietary intake of refined sugar with diabetes [1]. In line with these findings, in 1929, Banting also hypothesized that the intake of refined sugar may be a major cause of adult-onset diabetes [2]. However, similar to the ongoing discussion nowadays (also see [3, 4]), others suggested that the increase in obesity and the prevalence of diabetes type 2 may simply result from overnutrition [5, 6]. Following this hypothesis which is based on the first law of thermodynamics, weight gain would simply result from the imbalance of energy ingestion vs. energy expenditure. Supporting the latter, at the time food became more plentiful and easier to obtain. Along with the introduction of new inventions like elevators and cars but also machines in industrial production leading to an easier ‘avoidance’ of physical activity, lifestyle started to change dramatically. On the other hand, results from epidemiological [7–10] and intervention studies [11–14] as well as animal experiments [15, 16] provide evidence that the consumption of certain foods like sugar-sweetened beverages may impact the development of overweight and metabolic abnormalities like hypertension, insulin resistance, dyslipidemia, and non-alcoholic fatty liver disease (NAFLD) irrespective of overnutrition. Some more recent studies reported that the intake of fructose enriched diets may even in the absence of marked weight gain be causative in the development of metabolic abnormalities [14, 17]. Results of controlled intervention trials also suggest that glucose and fructose may not only differently affect glucose and insulin release, but may also affect gut microbiota composition and intestinal barrier function as well as the release of gastrointestinal hormones [18–23]. Some of the so far accumulated data are rather contradictory; still, all in all, data so far fuel the assumption that different sugars may impact health differently. Starting from this background, the aim of the present narrative review is to provide some insights in dietary intake and metabolism of fructose and to summarize recent as well as older findings assessing the effects of fructose on intestinal microbiota, homeostasis, and barrier function. Furthermore, these findings are related to the development of metabolic diseases with a specific focus on the liver.

## Dietary intake of fructose

Fructose is naturally found in fruits, vegetables (e.g., in peppers or carrots), or honey. In addition, added sugars, like sucrose or high-fructose corn syrup (HFCS), also contribute markedly to the dietary intake of fructose [24] (for overview see Table 1). Nowadays, HFCS is being used in many countries as a replacement of sucrose in foods and beverages such as soft drinks, sweets, bakery goods, and dairy products. To produce HFCS, some of the glucose found in corn syrup is enzymatically converted to fructose [23, 25]. With implementing the latter technique in the USA in the 1970s, sucrose intake markedly decreased in the USA, while concurrently HFCS consumption increased resulting at first in a rather stable overall sugar intake [25]. Results of epidemiological studies suggest that from 2001 to 2021 the total daily intake of HFCS decreased by almost 40 % from 46 g/d to 28 g/d in the USA (data from US Department of Agriculture) [26]. Despite changes in the sugar market in 2017 [27], in most European countries, HFCS is not widely used and the intake of HFCS is, compared to the USA, rather low [28]. Indeed, sucrose is still the main added sugar found in foods and beverages in Europe [29]. And while there were also some decreases in average sugar intake in several European countries as well as in South- and Central American countries and in Australia, added sugar intake in most of these regions is still above the recommendations of the World Health Organization (WHO; [30]; energy derived from added sugar intake <10 % of total energy intake accounting to ~50 g of sugar in a 2,000 kcal diet [31]). For instance, surveys conducted in European and Latin American countries report that total sugar intake (= sucrose intake) in adults accounts for ~15–21 % of the total energy intake [32]. Results of studies also suggest that socio-economic status and the intake of added sugars negatively correlate with lower income households shown to have a markedly higher sugar intake [33].

**Table 1:** Fructose, glucose, and sucrose content of various foods and beverages

Food	Free fructose [g]/100 g	Total fructose [g]/100 g^a^	Free glucose [g]/100 g	Total glucose [g]/100 g^a^	Sucrose [g]/100 g
Caffeinated beverages	0.0	5.3	0.0	5.3	10.6
Energy drinks	0.0	5.5	0.0	5.5	11.0
Lemonades	0.5	5.6	0.5	5.6	10.1
Cake	0.9	5.3	1.1	5.5	8.7
Cake with fruits	4.6	11.9	1.7	9.0	14.5
Cookies	0.1	25.2	0.1	25.2	50.2
Fruit ice cream	1.7	13.5	0.6	12.4	23.6
Honey	38.8	40.0	33.9	35.1	2.4
Apple	5.7	7.0	2.0	3.3	2.5
Banana	3.4	8.6	3.5	8.7	10.3
Grape	7.1	7.3	7.1	7.3	0.4
Asparagus	0.9	1.0	0.8	0.9	0.2
Carrot	0.8	3.3	0.8	3.3	4.9
Pepper	1.1	1.2	1.3	1.4	0.1
Apple juice	6.4	7.3	2.4	3.3	1.7
Orange juice	2.5	4.2	2.6	4.3	3.4
Yoghurt (plain)	0.0	0.0	0.0	0.0	0.0
Yoghurt (with fruits)	0.3	5.4	0.2	5.3	10.1

Data from ‘Österreichische Nährwerttabelle’ based on Bundeslebensmittelschlüssel, Austria

^a^Total fructose/glucose derived from sucrose and free fructose/glucose

Studies assessing fructose rather than sugar intake are rather limited. Results of our own studies and those of others suggest that the average fructose intake of healthy middle-aged individuals in Germany and Austria ranges from ~40 g to ~49 g fructose/d ([14, 34–36] and unpublished data). Data from the Dutch National Food Consumption Survey 2007–2010 also revealed a similar dietary daily fructose intake in Dutch population (7–69 years, average fructose intake ~46 g/d with 67 % consumed as sucrose and 33 % as free fructose with the main food sources being soft drinks, juices, cakes and cookies) [37]. Studies from Brasilia suggest an average intake of 32 g (for women) to 35 g (for men) of total fructose per day mainly consumed as soft drinks or fruit juice. In Lebanon, daily intake of fructose seems to be on average 51 g/d. In this study, it was also estimated that the intake of added fructose stemming from HFCS was three times higher than the intake of naturally occurring fructose in fruits and vegetables [38, 39]. Taken together, these data suggest that free sugar intake in general and free fructose intake in particular are still markedly higher in wide parts of the general population than recommended in many countries world-wide.

## Uptake of fructose in small intestine and fructose metabolism

As fructose and glucose uptake and metabolism are quite different and as these differences are discussed to be at least in part contributing to the different (patho-) physiological effects of these two monosaccharides, in the following, some of the key differences regarding the uptake and the metabolism of fructose are summarized.

### Uptake of fructose in small intestine

**Fig. 1:**
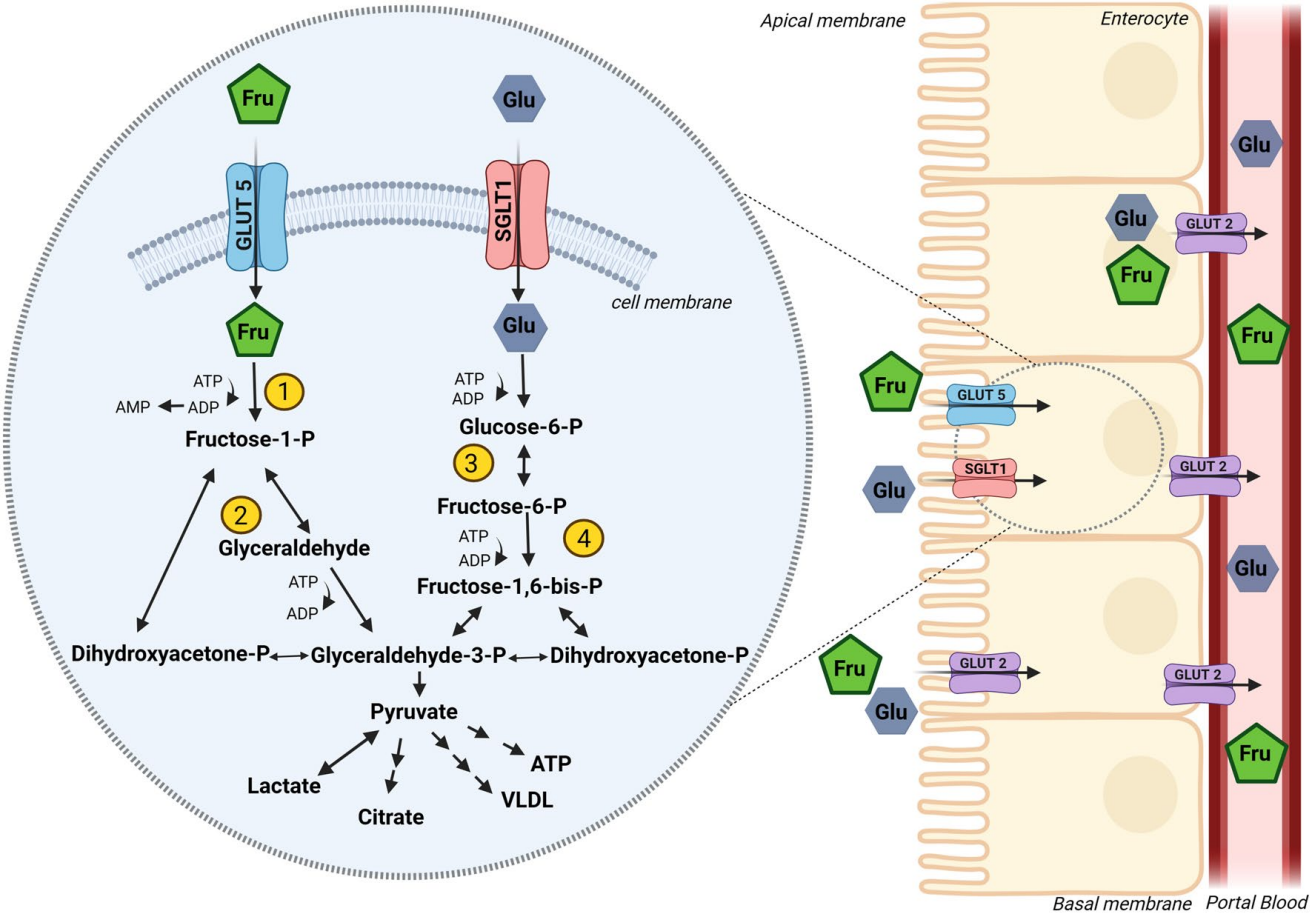
Fructose (Fru) and glucose (Glu) uptake and metabolism in enterocytes. At the apical side of the enterocytes, glucose is taken up via sodium-dependent glucose transporter 1 (SGLT1), whereas fructose is taken up via glucose transporter 5 (GLUT5). At high concentrations of luminal saccharides, GLUT2 may also contribute to the apical uptake of glucose and fructose into enterocytes. Fructose is metabolized to fructose-1-phosphate via ketohexokinase (KHK) (1) and is further converted to dihydroxyacetone phosphate and glyceraldehyde-3-phosphat via aldolase B (2), which can be processed to pyruvate being a key molecule for the production of lactate, citrate, adenosine triphosphate (ATP) or very low-density lipoprotein (VLDL). In contrast, glucose is metabolized to fructose-6-phosphat via the enzyme glucose-6-phosphate isomerase (3) and further converted to fructose-1,6-bisphosphate through the enzyme phosphofructokinase 1 (4). At the basolateral side of the enterocyte, both sugars are released into the portal blood via GLUT2 for both, glucose and fructose. Figure was created with BioRender.com and modified from [40].

It is well described that the uptake of fructose and glucose in small intestine differs (see [40] and also Fig. 1). As first described by Crane in 1962 [41], glucose is mainly taken up actively into the enterocytes through an energy- and sodium-dependent transporter, the so-called sodium-dependent glucose transporter (SGLT1) (see [35, 36]). When luminal glucose concentrations are high, it has been shown that the glucose transporter 2 (GLUT2) is rapidly and transiently recruited to the apical enterocyte membrane [42], suggesting that GLUT2 also facilitates some of the uptake of glucose. In contrast, fructose is taken up into the enterocytes via the energy-independent glucose transporter GLUT5 (see [35, 36]). Supporting the assumption that GLUT5 is the main apical fructose transporter in small intestinal enterocytes, results of *ex vivo* studies employing intestinal tissue obtained from GLUT5 knockout mice showed no uptake of fructose, despite normal GLUT2 levels [43]. However, results of *in vivo* and *ex vivo* studies also suggest that under certain conditions—like after the intake of high fat and/or sugar (fructose) diets—luminal fructose uptake into enterocytes may also be facilitated through GLUT2 [43–45]. Studies further suggest that concentrations of GLUT5 in enterocytes can be induced when fructose is present in the intestinal lumen in wildtype mice [46]. Interestingly, concentrations of other GLUT transporters like GLUT7, 8, and 12 were not altered by the presence of fructose [46]. On the other hand, as reviewed in detail by Iametti et al., studies suggest that intestinal fructose uptake may be reduced when polyphenols are concomitantly present [47]. For instance, it has been shown in *in vitro* studies employing Caco-2 cells that polyphenols like apigenin and derivatives of apigenin, found for instance in chamomile tea, but also catechins found in green tea, can reduce fructose uptake dose-dependently up to ~70 % [48]. In these studies, it has also been shown that some of these compounds may interfere with GLUT5 mRNA expression while others seem to block GLUT5 through other yet not fully understood measures. It has been shown that fructose-dependent regulation of GLUT5 expression and recruitment to the apical membrane of enterocytes is at least in part regulated through PI3 kinase/Akt-dependent signaling pathways [49]. Furthermore, it has also been shown that a low glycemic diet may inhibit fructose uptake via GLUT2 through alterations of the translocation of GLUT2 into the apical membrane [44].

At the basolateral side of the enterocytes, export of both fructose and glucose into the blood has been shown to be mediated through GLUT2 (see [35] and also Fig. 1). Still, even after an elevated intake, fructose concentrations in peripheral blood remain rather low. Indeed, in fasting serum of healthy subjects, levels are ~8.1 µM [50, 51], suggesting that fructose, at least in part, is metabolized by enterocytes. Supporting the latter idea, Bode et al. reported already in the 80s that fructose intake compared to glucose or starch consumption contributes to adaptive changes of enzymes involved in fructose metabolism in jejunal mucosa of rats [52]. In recent years, Jang et al. [53] showed in mice that a gavage of low doses of fructose (0.5 g fructose/kg body weight) was almost completely cleared by enterocytes (~90 %) via fructokinase. In the same study, it was also shown that when ingested in doses >1 g fructose/kg body weight, fructose reaches the liver (~30 %) and colonic microbiota, respectively [53]. Studies in rodents further reported that in the absence of fructose intake, fructose levels are <0.1 mM in portal and systemic blood [50]. In contrast, in mice, an increase in fructose levels in systemic blood of 0.2 to 1 mM has been shown after ingesting a fructose enriched diet (20-40 % fructose) [50]. In line with the hypothesis that marked amounts of fructose are metabolized in the enterocytes of the small intestine, studies in hamsters showed an induction of intestinal *de novo* lipogenesis and apoprotein B48 synthesis after dietary fructose intake [54]. Studies with healthy volunteers employing isotope labeled fructose showed that only ~4 g of a 30 g fructose load reached systematic circulation [55]. However, whether this is related to a very effective clearance of fructose by the liver or to a metabolism of the monosaccharide in enterocytes has not yet been clarified.

### Metabolism of fructose

Within cells, metabolism of fructose and glucose also differs substantially, which has been reviewed in great detail by others (see [34, 56] and Fig. 1). In brief, fructose is phosphorylated to fructose-1-phosphate through the enzyme fructokinase C using adenosine triphosphate (ATP) as a co-substrate (see [57]). The monosaccharide is then metabolized via aldolase B to dihydroxyacetone phosphate (DHAP) and glyceraldehyde. Glyceraldehyde is converted to glyceraldehyde-3 phosphate. Enzymes necessary for these metabolic steps have only been shown to be expressed in enterocytes, hepatocytes, and proximal tubular cells [56]. From there on, the metabolisms of fructose and glucose are alike [56, 58].

The conversion of fructose to fructose-1-phosphate is facilitated in the absence of any feedback control thereby contrasting the metabolism of glucose being tightly regulated (see [34] and Fig. 1). Subsequently, intermediates of the metabolism of fructose such as glyceraldehyde-3-phosphat and DHAP are built without regulation bypassing phosphofructokinase. The latter enzyme is the major regulatory step of glycolysis (see [56, 59]). Also, converting fructose to fructose-1-phosphate requires ATP as a co-substrate [60]. Studies have shown that in settings of high fructose intake, ATP can be depleted resulting in an activation of adenosine monophosphate (AMP) deaminase, and subsequently, an induction of the purine nucleotide turnover and the production of uric acid [61]. Interestingly, the decrease in ATP levels following a fructose challenge has been shown to last for up to 50 min thereby outlasting the increase in blood fructose levels [62]. These findings suggest that other reactions besides the phosphorylation of fructose may contribute to the decrease in ATP levels in liver tissue [63]. Further studies are needed to determine associated molecular mechanisms. Also, it remains to determine if this temporary fructose-induced depletion of ATP is critical in the development of liver diseases like NAFLD (also see below).

## Fructose, insulin resistance and NAFLD: role of bacterial endotoxin and Toll-like receptors

Despite intense research efforts throughout the last decade, mechanisms underlying the metabolic alterations associated with the intake of elevated amounts of fructose, and especially, the development of insulin resistance and associated diseases like NAFLD are not yet fully understood. As detailed above, due to its unregulated metabolism, fructose may be converted to various metabolites like diacylglycerol rather fast. Results of some animal studies suggest that hepatic insulin resistance and steatosis may result from an increase in hepatic diacylglycerol accumulation, and an associated protein kinase C activation leading to alterations of insulin-mediated Akt activation [64]. However, as discussed above, several studies assessing the uptake and distribution of fructose suggest that marked amounts of fructose may already be metabolized in the small intestine ([53] and Fig. 1). Accordingly, other (additional) mechanisms may be involved in the development of insulin resistance associated with an elevated fructose intake and NAFLD. In support of this assumption, results of animal studies of our own group have shown that a chronic intake of a fructose enriched drinking solution (30 % of fructose content) and the resulting development of fatty liver are associated with increased bacterial endotoxin levels in portal vein and an induction of its receptor, Toll-like receptor 4 (TLR4), in the liver as well as its subsequent signaling cascade [15]. In the same study, it was also shown that alterations alike are not present when animals are fed a 30 % glucose solution. Furthermore, the concomitant treatment of mice with non-resorbable antibiotics like polymyxin B and neomycin abolished the increase in bacterial endotoxin levels in portal vein being also associated with a diminishment of the development of liver steatosis and inflammatory alterations in mice [15]. Bacterial endotoxin and an activation of TLR4 signaling have also been shown to contribute to the development of insulin resistance [65, 66]. Supporting the hypothesis that an elevated fructose intake may impair intestinal barrier function, resulting in an increased translocation of bacterial endotoxin, mice lacking a functional TLR4 were found to be significantly protected from fructose-induced liver damage [67]. Also, targeting alterations of intestinal barrier function with drugs like metformin or bile acids and probiotics (also see below), respectively, have been reported by us and others not only to be associated with ‘normalized’ tight junction protein levels in small intestine but also with a lessening of the development of NAFLD and a normalization of markers of insulin resistance in liver tissue [68–72]. Interestingly, in our studies only limited or no effects on tight junction proteins were found in colon [73]. These findings suggest that alterations associated with the intake of elevated amounts of fructose may not only result from changes of intestinal microbiota composition but also may result from direct effects of fructose, e.g., its metabolism, in enterocytes. In line with these findings, Guo et al. showed that the chronic intake of fructose in piglets decreased the expression of tight junction proteins and myosin light chain kinase (MLCK) in ileal tissue [74]. Wagnerberger et al. further reported that not only TLR4 is induced in livers of mice fed a fructose-rich diet. Rather, in this study it was shown that the expressions of other TLRs including TLR1, 2, 3, and 6-8 mRNA were all significantly higher in mice fed a fructose-rich diet than in controls. Furthermore, expression of TLRs in the liver was almost completely abolished when fructose-fed mice were concomitantly treated with the non-resorbable antibiotics polymyxin B and neomycin. Interestingly, while the disruption of TLR4 signaling or the treatment with non-resorbable antibiotics was associated with a reduction in markers of insulin resistance and inflammation, e.g., number of F4/80 positive cells, inducible nitric oxide synthase (iNOS), and tumor necrosis factor alpha (TNFα) expression, fat accumulation in liver tissue was only reduced by ~50–60 % [73]. These results further suggest that some of the fructose may reach the liver and may through its insulin-independent metabolism be quickly converted to triglycerides.

**Fig. 2:**
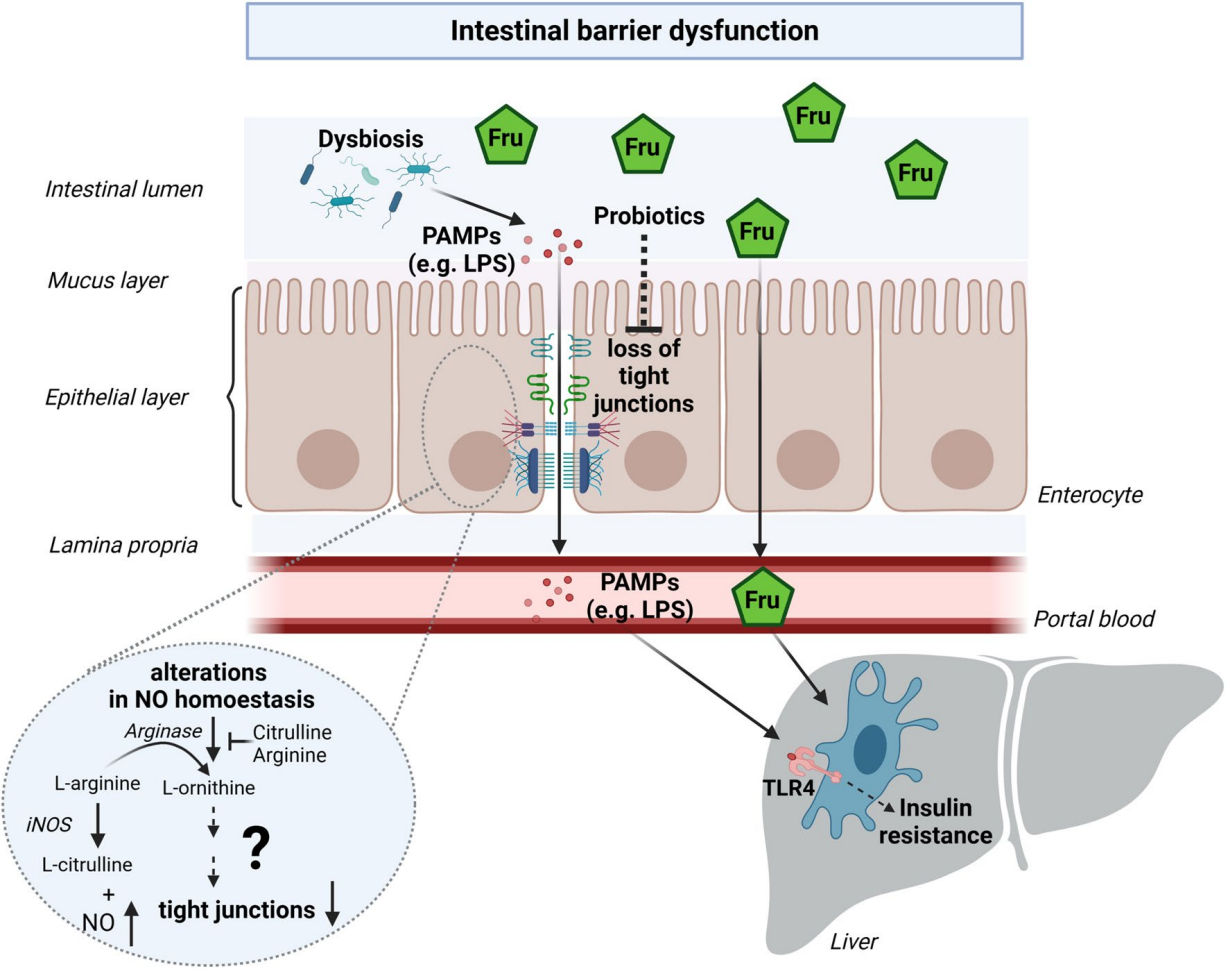
Schematic drawing of possible mechanisms underlying high fructose-induced intestinal barrier dysfunction. High fructose (Fru) consumption may lead to changes in microbiota composition (dysbiosis). Furthermore, elevated fructose intake can induce intestinal barrier dysfunction, e.g., a loss of tight junction proteins, subsequently leading to an increased permeation of pathogen-associated molecular patterns (PAMPs). Higher translocation of PAMPs like lipopolysaccharides (LPS) into the portal vein can further lead to an induction of Toll-like receptor (TLR4) receptor-dependent signaling cascades in the liver which may contribute to the development of hepatic insulin resistance. Moreover, fructose metabolism may, through yet to be determined mechanisms, directly affect mucosal nitric oxide (NO) homeostasis (e.g., shift in NOS and arginase activity: increased formation of NO and low arginase activity) leading to a loss of tight junction proteins. The amino acids L-arginine and L-citrulline may attenuate the decrease in arginase activity and therefore, may dampen fructose-induced intestinal barrier dysfunction. Some probiotics may also attenuate the development of fructose-induced intestinal barrier dysfunction through mostly unknown mechanisms. Figure was created with BioRender.com.

In line with the above summarized findings in animal studies, it has been shown in human studies that a three day long elevated intake of fructose (25 % of total energy as fructose) is associated with increased bacterial endotoxin levels and an induction of TLR2 and 4 mRNA expression in peripheral blood mononuclear cells in healthy volunteers [14]. In the same study, similar alterations were not found when the same subjects consumed comparable amounts of glucose for three days. In summary, these findings suggest that both intermediates of the hepatic fructose metabolism and pathogen-associated molecular patterns (PAMPs) and -dependent signaling cascades may contribute to the development of fructose-associated insulin resistance and the development of NAFLD (see Fig. 2). However, further studies are needed to determine doses and to further delineate molecular mechanisms. Some of the so far defined possible mechanisms underlying the increased translocation of PAMPs are highlighted in the following.

## Fructose, intestinal microbiota and intestinal barrier

### Fructose and intestinal microbiota composition

Alterations of intestinal microbiota composition have repeatedly been discussed to be associated with impairments of intestinal barrier function and an increased translocation of PAMPs like bacterial endotoxin [75]. Furthermore, results of studies in rats and mice suggest that a chronic intake of fructose-rich diets either feeding fructose alone or in combination with a high-fat diet is associated with marked changes in the relative abundance of several bacterial families and species in feces [76, 77]. Specifically, results of several studies suggest that a chronic intake of a fructose-rich chow or drinking solution results in a decrease in *Bifidobacterium* and *Lactobacillus* in feces of rats [78, 79]. Furthermore, Wang et al. reported that in mice, the intake of a fructose- and fat-rich diet resulted in an increase in the ratio of *Firmicutes* to *Bacteroidetes* and *Lactobacillus*, uncultured bacterium *Erysipelotrichaceae*, *Olsenella,* and uncultured bacterium *Bacteroidales S24-7* group as well as the relative abundance of *Desulfovibrio*, *Blautia*, *Catenibacterium*, *Bacteroides*, *Candidatus Saccharimonas,* and *Faecalibaculum* in feces [80]. In a study employing Kunming mice, the chronic intake of a 30 % fructose solution was associated with a decrease in the relative abundance of *Bacteroidetes,* while that of *Firmicutes* was increased [81]. These changes also resulted in an increase in the ratio of *Firmicutes* to *Bacteroidetes* in feces of fructose-fed animals [81]. In another study, in which mice were fed a 60 % fructose solution, microbial composition generally changed being associated with a pronounced decrease in the *Bacteroidetes*/*Firmicutes* ratio as well as an increase in the relative abundance of *Bacteroides*, *Akkermansia*, *Lactobacillus*, and *Ruminococcus* in feces [82]. In line with these findings, it was recently reported that in fructose-fed piglets the ratio of *Firmicutes* to *Bacteroidetes* in colon was also increased [74], further suggesting that the alterations found regarding intestinal microbiota might be species independent. Also, in this study, the relative abundance of *Blautia* and *Clostridium sensu stricto 1* were higher than in piglets fed the control diet [74]. Supporting the hypothesis that chronic high intake of fructose may affect the host through alterations of the intestinal microbiota composition, studies indicate that in rats fed a high-fructose diet levels of short-chain fatty acids in plasma are reduced [83].

Earlier studies reported only limited effects on microbiota composition in the upper part of small intestinal tissue in mice fed a 30 % fructose solution probably due to a lack of specification of the method [68]. In contrast, recent studies employing 16S rRNA Illumina sequencing technologies revealed that an intake of a fructose-rich diet (e.g., fructose alone or in combination with fat) over an extended period of time is associated with marked changes in overall diversity of intestinal microbiota and relative abundance of specific bacterial families and species in ileum. For instance, Guo et al. reported that a chronic intake of fructose in piglets was associated with higher Chao and Shannon indexes in ileum when compared to controls. Furthermore, in the same study, the proportion of *Firmicutes* and *Proteobacteria* decreased, but the proportions of *Bacteroidetes*, *Actinobacteria* and *Tenericutes* increased [74].

Taken together, results of animal studies suggest that a chronic intake of large amounts of fructose is associated with a shift in the ratio of *Firmicutes* to *Bacteroidetes* and the relative abundance of some bacterial species in lower parts of the gut, e.g., the ileum and colon as well as feces; however, while it has been suggested by some studies, that this may also impact the metabolite pattern, it has not yet been clarified if the ‘dysbiosis’ imposed by the consumption of high amounts of fructose over an extend period of time also impacts the host by other means. Also, specific effects seem to vary between species and among mouse strains as well as fructose doses and feeding duration. Furthermore, in most studies no direct links and/ or mechanisms underlying the increased translocation of PAMPs in the gut or induction of TLRs and -dependent signaling cascades in liver and other tissues in settings of high fructose intake have been established. In summary, while several studies suggest that a high fructose intake may trigger gut microbiota dysbiosis in both feces and small intestine, the impact of this ‘dysbiosis’ on the host required further studies to fully understand the impact of these alterations.

### Fructose and intestinal barrier dysfunction: Alterations of NO homeostasis

As discussed above, results of studies suggest that marked amounts of dietary fructose may already be metabolized in small intestinal enterocytes also suggesting that fructose could either directly or through intermediates affect these cells. Indeed, employing an *ex vivo* model of small intestinal everted tissue sacs, we recently showed that even in the absence of bacteria physiological concentrations of fructose (5 mM) may alter intestinal barrier function in as short as 30–60 min upon exposure [84]. Results of these studies also suggest that alterations of the nitric oxide (NO) homeostasis may be critical herein [84, 85]. Specifically, it has been shown by us and others that chronic intake of fructose is associated with an induction of iNOS and NO synthesis in the gut [86, 87]. And while results of our own group also showed that iNOS knockout mice are not protected from the increased translocation of bacterial endotoxin into the portal blood stream [86], targeting NO production in small intestinal tissue, e. g., through L-arginine or L-citrulline (also see below) has been shown to dampen fructose-induced intestinal barrier dysfunction [84, 88]. Supporting the hypothesis that alterations of the NO homeostasis are critical in fructose-induced intestinal barrier dysfunction, an oral supplementation of L-arginine and L-citrulline, respectively, has been shown to attenuate the loss of intestinal barrier function (e.g., the loss of tight junction proteins and increased permeation of xylose) [84, 88]. This was also associated with a lower translocation of endotoxin into the portal vein in various rodent models employing fructose-rich diets (30 % fructose in drinking water [89]; 50 % wt/wt fructose in liquid diet [90, 91]). The protective effects of the amino acids were associated with ‘normalization’ of arginase activity found to be markedly lower in small intestinal tissue of mice-fed fructose enriched diets and in *ex vivo* models of small intestinal everted tissue sacs challenged with fructose [84, 88]. These findings are in line with those of others reporting a critical role of arginase in inflammation in intestinal tissue [92, 93]. L-arginine and L-citrulline have both been shown to be allosteric regulators of arginase activity. Interestingly, the supplementation of L-citrulline had no effect on the alteration of intestinal microbiota composition in small intestine inflicted by the feeding of a fructose-, fat- and cholesterol-rich diet. Furthermore, a treatment of animals with the arginase inhibitor nor-NOHA attenuated the protective effects of L-arginine and L-citrulline, respectively, being also associated with an attenuation of the protective effects of the two amino acids on the development of NAFLD [84, 88]. Results of our own studies also suggest that the ‘normalization’ of arginase activity was associated with a decrease in MLCK protein [84]. It has been shown that MLCK activity may be induced in cells treated with spermine [94], the latter being a downstream substrate of arginase-mediated formation of ornithine. However, if the lower arginase activity found in small intestinal tissue exposed to fructose alters spermine bioavailability, and hereby, MLCK activity remains to be determined. Also, despite the results of studies of our own group suggesting that arginase activity is also lower in patients with steatosis, further studies are needed to determine if this reduction in arginase activity is related to an increased fructose intake and if this is causal in the increased bacterial endotoxin levels found in these patients [84]. Also, the question how fructose alters NO homeostasis remains to be answered.

## Fructose and effects of probiotics on intestinal barrier and liver health

As detailed above, results of several studies suggest that an intake of large amounts of fructose can alter intestinal microbiota composition and barrier function. These results tempt the assumption that a manipulation of intestinal microbiota composition through the supplementation of probiotics may alter the fructose-induced alterations. Indeed, as detailed in the following, results of several studies suggest that a concomitant intake of probiotics may attenuate at least some of the effects of fructose. For instance, the concomitant treatment of fructose-fed mice with the probiotic *Lactobacillus casei Shirota* significantly diminished liver damage compared to mice only fed fructose [68]; interestingly, this was not associated with a protection from the loss of tight junction proteins or changes of intestinal microbiota composition in small intestine. Ritze et al. and Zhao et al. both reported that a concomitant intake of the probiotic *Lactobacillus rhamnosus GG* markedly attenuated the development of fructose-induced liver damage in mice [95, 96]. The protective effect of these probiotics was associated with a protection against the loss of tight junction proteins and impairments of intestinal barrier function. Also, *Lactobacillus rhamnosus GG* has been reported to ‘revert’ intestinal dysbiosis and decreased the relative abundance of inflammation-related bacteria such as *Desulfovibrionaceae*, *Clostridia,* and *Proteobacteria* in feces of fructose-fed animals [81]. The probiotic strains *Lactobacillus plantarum ATG-K2* and *ATG-K6* but also *Lactobacillus plantarum* strains such as *NA136* have also been shown to ameliorate the induction of pro-inflammatory markers like TNFα and interleukin-6 as well as markers of lipogenesis, e.g., fatty acid synthase and sterol regulatory element-binding protein 1c in small intestinal tissue of rodents fed a fat- and fructose-rich diet [97, 98]. Furthermore, in the study of Wang et al. the concomitant treatment of mice fed a fat- and fructose-rich diet with the probiotics *L. rhamnosus LS-8* and *L. crustorum* MN047 attenuated the development of NAFLD, insulin resistance, and decreased circulating lipopolysaccharides (LPS) levels [80]. It has also been shown that treating high fructose diet-fed mice with the bacterial strain called *Lactobacillus brevis DM9218* resulted in an improved intestinal barrier function combined with a reduction in LPS in the liver [99]. It has further been reported that the administration of the probiotic *Lactobacillus kefiri* and *Lactobacillus fermentum CECT5716* may diminish the effects of high fructose-induced dysbiosis [100, 101].

Taken together, these data further suggest that some probiotics may attenuate or at least diminish fructose-induced alterations of intestinal barrier function, and subsequently, the development of metabolic diseases like NAFLD. However, the above summarized data also suggest that not all beneficial effects on the development of fructose inflicted NAFLD found for probiotics may be related to an improved intestinal barrier function. Rather, results so far suggest that depending on the bacterial strain some of the probiotics might—probably through metabolites—alter liver metabolism directly while others seem, either through direct interaction or yet to be defined metabolites/mechanisms, to alter metabolism or signaling cascades within the enterocytes. Further studies are at need to determine mechanisms and metabolites involved.

## Conclusion

High fructose consumption, be it through food and beverages sweetened with sucrose or HFCS, may not only lead to the development of overweight but may also contribute to the development of other metabolic diseases like diabetes type 2 and NAFLD. Despite intense research effort, mechanisms underlying these alterations associated with fructose and their contribution to the development of the latter disease are not yet fully understood. Studies suggest that fructose may affect intracellular signaling through direct measures due to insulin-independent metabolism [40]. However, in recent years, it has also been shown in various species that a high and chronic intake of fructose can be associated with changes in fecal microbiota but also a loss of tight junction proteins in small intestine. A causal link between the changes in fecal microbiota composition and the loss of tight junctions in small intestine could be established based on the findings employing certain probiotics like *Lactobacillus rhamnosus GG* and *Lactobacillus brevis DM9218* that have been shown to attenuate the development of intestinal barrier dysfunction [96, 99]. However, results of recent other studies also suggest that fructose may add to intestinal barrier dysfunction and the subsequent translocation of PAMPs like bacterial endotoxin through more direct interaction with the enterocytes and their metabolism. Indeed, it has been shown that fructose metabolism directly disturbs intracellular NO homeostasis, e.g., leading to a loss of tight junction proteins ([84, 88] and see Fig. 2).

Further studies are needed to determine mechanisms and doses necessary to inflict these alterations. Also, studies are needed to elucidate the impact of intestinal microbiota and the interaction with other compounds found in foods and beverages like polyphenols herein. It remains yet to be determined if and how specific (probiotic) bacterial strains can alter or even attenuate these alterations. As most of the results showing an effect of fructose on intestinal microbiota composition and barrier function were obtained in *in vitro* and animal studies, studies in humans are needed in the future to determine if alterations alike are also found in humans and if so, whether these alterations contribute to the development of intestinal barrier dysfunction, and subsequently, the development of metabolic diseases like NAFLD and diabetes type 2. Moreover, whether sex- or age-specific differences have an impact on alterations associated with high fructose intake has not yet been determined to our knowledge. Despite these open questions, and especially when taken the high intake of sugar and fructose found in some populations into consideration, a restrictions of sugar intake, and herein, especially fructose intake may already now be a way to lessen intestinal barrier dysfunction and the development and progression of metabolic diseases associated with altered intestinal barrier function like NAFLD. Indeed, in recent years, guidelines have been issued and different political measures including restrictions of the promotion for sugar-rich products like sweets and sugar-sweetened beverages, the introduction of progressive taxes on sugary drinks and foods, and restrictions of the availability of specific foods have been established in many countries all aiming to reduce sugar intake [102]. Also, changing food choice settings, e.g., removing sugary snacks and beverages from near tills to reduce impulse buying and improving nutritional literacy especially in children, adolescents, and young adults through new didactic tools in school settings may also add to reduce overall sugar intake, and thereby, positively impacting on health outcomes in the general population.

## Data Availability

Not applicable as there were no new data generated.
